# Internet-Administered Emotional Awareness and Expression Therapy for Somatic Symptom Disorder With Centralized Symptoms: A Preliminary Efficacy Trial

**DOI:** 10.3389/fpsyt.2021.620359

**Published:** 2021-02-12

**Authors:** Daniel Maroti, Josefine Ek, Rose-Marie Widlund, Howard Schubiner, Mark A. Lumley, Peter Lilliengren, Indre Bileviciute-Ljungar, Brjánn Ljótsson, Robert Johansson

**Affiliations:** ^1^Department of Clinical Neuroscience, Karolinska Institute, Stockholm, Sweden; ^2^Department of Psychology, Uppsala University, Uppsala, Sweden; ^3^Department of Internal Medicine, Ascension Providence-Providence Hospital, Michigan State University College of Human Medicine, Southfield, MI, United States; ^4^Department of Psychology, Wayne State University, Detroit, MI, United States; ^5^Department of Health Care Sciences, St. Lukas Educational Institute, Ersta Sköndal Bräcke University College, Stockholm, Sweden; ^6^Department of Clinical Sciences, Karolinska Institutet and Multidisciplinary Pain Unit, St Göran Hospital, Stockholm, Sweden; ^7^Department of Psychology, Stockholm University, Stockholm, Sweden

**Keywords:** emotional awareness and expression therapy, fibromyalgia, irritable bowel syndrome, pain, self-help, emotional processing, trauma, somatic symptom disorder

## Abstract

**Background:** There is growing evidence that trauma, psychosocial conflict, and difficulties with emotional processing contribute to centralized somatic symptoms. Emotional Awareness and Expression Therapy (EAET) was developed to address these factors and reduce symptoms, and EAET has shown efficacy in face-to-face formats. No trial of an internet-delivered EAET (I-EAET) exists, however, so we developed such an intervention and conducted an uncontrolled feasibility and potential efficacy trial of I-EAET for patients with Somatic Symptom Disorder (SSD) with centralized symptoms (SSD-CS).

**Method:** After screening potential participants, a sample of 52 patients (50 women, two men; age *M* = 49.6, *SD* = 11.9) diagnosed with SSD-CS initiated treatment. I-EAET consisted of nine weekly modules focused on psychoeducation, emotional awareness and exposure, and anxiety regulation with self-compassion. Therapists communicated with each patient by email for about 20 min per week during treatment, answering questions and giving feedback on homework assignments. Patients completed measures of somatic symptoms, depression, anxiety, trauma-related symptoms, and functional disability before treatment and again at post-treatment and 4-month follow-up.

**Results:** A large reduction in somatic symptoms (PHQ-15) occurred pre-to post-treatment (*d* = 1.13; 95% CI: 0.84–1.47) which was fully maintained at 4-month follow-up (*d* = 1.19; 95% CI: 0.88–1.56). Twenty-three percent of the patients at post-treatment and 27% at follow-up achieved a 50% or greater reduction in somatic symptoms, and about 70% achieved a minimally important clinical difference. In addition, at post-treatment, there were small to medium reductions (*d*'s from 0.33 to 0.72) in anxiety (GAD-7), depression (PHQ-9), trauma-related symptoms (PCL-5), and functional disability (Sheehan Disability Scale). For all of these secondary outcomes, improvements were slightly to substantially larger at follow-up than at post-treatment (*d*'s from 0.46 to 0.80).

**Conclusion:** I-EAET appears to be a feasible treatment for adults with SSD and centralized symptoms, resulting in substantial and durable improvement not only in somatic symptoms but in other psychiatric symptoms and functioning. Controlled trials are needed determine the effects of I-EAET specifically and how this approach compares to face-to-face EAET and to other internet-delivered treatments, such as cognitive-behavioral interventions. Research should also identify treatment responders and mechanisms of change in EAET.

**Clinical Trial Registration:** www.ClinicalTrials.gov, identifier: NCT04122846.

## Introduction

In 1895, Breuer and Freud wrote about Anna O, a patient who, among other somatic symptoms, suffered from severe pain and was bedridden for months (1). Her condition was believed to be psychological rather than neurological in origin, and hence she was treated with what she herself named the “talking cure.” Since the time of Anna O., persistent physical symptoms presumably due to psychological factors have gone by many different names: medically unexplained symptoms (MUS), functional somatic syndromes (FSS), somatoform disorders (SD), bodily distress syndrome, and others. However, concerns over mind-body dualism and difficulties ruling out disease processes (2) led to the development of Somatic Symptom Disorder (SSD) in the DSM-5 (3). SSD is characterized by one or more chronic somatic symptoms (e.g., chronic bodily or head pain, abdominal symptoms, and fatigue) that are distressing or result in significant disruption of daily life, as indicated by disproportionate and dysfunctional cognitive, emotional, and behavioral responses. The prevalence of SSD is estimated to be 5–7% in the general population and 17% in primary care and has a very chronic course, with up to 90% of patients with SSD reporting symptoms lasting longer than 5 years (4).

Although terms such as “medically unexplained,” “functional,” or “psychogenic” are conceptually problematic and are falling from favor, the concept of “central sensitization,” or simply “centralization,” has gained scientific and clinical acceptance (5, 6). In this framework, the central nervous system is recognized as being primed by adverse life experiences and sensitized by bodily injury and pain to augment or amplify somatic symptoms or possibly even generate them (7–12). Such ppersistent centralized physical symptoms are both very prevalent and costly, imposing a heavy burden on the individual and society, with over one-third of primary care patients thought to have such symptoms (13). The presence of centralized physical symptoms is associated with psychiatric comorbidity as well as functional disability, such as unemployment or early retirement (14).

Cognitive behavioral therapy (CBT) is the most studied treatment for persistent centralized physical symptoms. Traditional CBT approaches teach patients cognitive and behavioral skills to manage their symptoms by changing unhelpful cognitions (e.g., by reappraisal), down-regulating arousal (e.g., by relaxation training), and increasing daily functioning (e.g., by activity pacing) (15–18). The effect size achieved by CBT on reducing persistent somatic symptoms tends to be small, whether CBT is delivered face-to-face or *via* the internet (19–21); for example, only about 13% of patients with fibromyalgia a have substantial symptom reduction following CBT (22).

One reason for the limited effects of CBT for SSD is that such approaches do not adequately address the impact of psychological trauma, psychosocial conflict, and subsequent difficulties with emotional processing that are common in patients with centralized somatic symptoms treatments (23). For example, a meta-analysis by Afari et al. (24) showed a 3-fold increased prevalence of psychological trauma in patients with functional somatic syndromes such as irritable bowel syndrome, fibromyalgia, or chronic fatigue syndrome compared to healthy controls. Studies have found prevalence's of post-traumatic stress disorder (PTSD) in up to 60% of patients with SSD (25). Emotional processing of trauma, including engaging in emotional awareness, differentiation, disclosure, and expression, is commonly disturbed in SSD (26). The suppression or avoidance of these emotional processes appears to be a core problem needing to be addressed in patients with persistent centralized physical symptoms such as some types of chronic pain (27) or FSS (28).

Lumley and Schubiner (23) developed Emotional Awareness and Expression Therapy (EAET) specifically to address the trauma and avoided emotional processing that plays an important role in centralized chronic pain and other persistent physical symptoms. EAET is an integrative therapy, using principles from modern affective and pain neuroscience, such as central sensitization and predictive coding of somatic perceptions, as well as theory and techniques from exposure-based, psychodynamic, and emotion-focused therapies. The rationale undergirding EAET is that centralized chronic pain and other somatic symptoms “can be rooted in, exacerbated, or maintained by unresolved stressful, traumatic, or conflictual emotional experiences” [(17), p. 2,361]. These emotional experiences lead to difficulties with emotional processing, which in turn cause or contribute to somatic symptoms. In EAET, therefore, a central focus is on exposure or emotional processing of avoided thoughts and feelings related to trauma or conflict. Such emotional processing, however, can be difficult for patients, who may have difficulty seeing connections between emotional processes and their symptoms. To overcome this problem, it is important to educate patients about the role of their brain (e.g., sensitization, predictive coding) and emotional processes in their symptoms. Moreover, techniques to self-soothe or regulate one's anxiety can be taught to help facilitate engagement in intense emotional exposure. Subsequently, activating patients' unexpressed adaptive emotions, particularly anger but also guilt, sadness, and love related to interpersonal experiences or relationships is a crucial step in EAET.

EAET in its current form has been evaluated and found to be superior to treatment as usual, education controls, or even CBT in five randomized controlled trials in patients with fibromyalgia (17), irritable bowel syndrome (29), pelvic pain (30), medically unexplained symptoms (31), and musculoskeletal pain (18). Although trials of EAET have included both individual and group formats, they have all been face-to-face, and no study has been conducted through the internet. Internet-administered, guided self-help has been found to be effective for many psychiatric conditions (32). Moreover, internet-delivered treatments have the advantage of reaching more patients, including those in rural areas, those who lack adequate transportation or other resources, who need flexible scheduling, or who are too ill to attend in person. Internet-delivered interventions also use less professional time, thereby saving resources, and have become increasingly valuable during the viral pandemic. Thus, we developed and tested in an uncontrolled trial an internet version of EAET, which we called I-EAET.

## Materials and Methods

### Eligibility Criteria

To be eligible for the study, a participant needed to be over 18 years of age, have an Internet connection, and fulfill criteria for DSM-5 SSD with a somatic symptom severity of > 10 on the PHQ-15, indicating at least moderate somatic symptoms and above the 75th percentile in the Swedish population (33). However, only patients with centralized somatic symptoms (23) where included in the current trial because EAET is designed to target centralized conditions, rather than physical symptoms based in somatic disease or structural pathology. Thus, patients were excluded if they had a somatic disease with recognized tissue damage (e.g., cancer, multiple sclerosis, or rheumatoid arthritis). Note that this exclusion criterion is a deviation from the DSM-5 diagnosis of SSD, which can include patients with a range of medical conditions. Hence, we labeled our sample as having SSD with centralized symptoms (SSD-CS).

DSM-5 SSD was diagnosed using the Health Preoccupation Diagnostic Interview [HPDI; (34)]. The Standard Mini International Neuropsychiatric Interview (M.I.N.I.; modules for eating disorders were excluded) was used to diagnose comorbid psychiatric disorders (35). Patients self-reported medical illnesses and diseases. To be included, patients needed to send in a certification from a doctor that acknowledged that the patient had persistent physical symptoms which effected psychological functioning, was likely to have an SSD diagnosis, and was judged as unlikely to get problematic side effects by participating in the study. Patients were included only if they explicitly endorsed an interest in investigating potential emotional factors contributing to their somatic symptoms. Data was collected on previous and ongoing medical and psychological treatments, and patients were excluded if they had other ongoing and interfering medical or psychological treatment, severe suicidal ideation, active bipolar disorder, or substance use disorder. No interference was judged to be the case if medications had been stable for 1 month and if psychological treatment was judged to be of a supportive nature.

### Procedures

Information about the study was published in a Facebook group for Swedish clinical psychologists in September 2019, asking for participants to join a study for “medically unexplained symptoms or somatic symptom disorder.” Those psychologists informed potential participants, who then expressed interest in the study through a website during the month that the enrollment was open. Written informed consent, baseline screening, and assessment of primary and secondary measures were conducted through a secure internet platform in October 2019. Psychiatric interviews (i.e., M.I.N.I and HPDI) were done by telephone during the same month. All interviewed participants were discussed at a multidisciplinary conference that included a senior physician specializing in pain and rehabilitation medicine (IBL); only after all of these steps was a patient considered potentially eligible for the study.

After being enrolled in the study, all participants were randomized to one of the therapists as their personal contact. Treatments started in November 2019, continued for 9 weeks, and ended in December 2019, when all post-treatment data was collected. Follow up was 4 months later, in April 2020. No monetary compensation was given, but participants received the treatment at no cost. This study was pre-registered at clinicaltrials.gov (NCT04122846) and received approval from the Swedish Ethical Review Authority (Approval number: 2019-03317).

### Treatment

This was a pilot study with a pre-test/post-test/follow-up design without a control group. The guided self-help treatment conducted through the Internet lasted for 9 weeks and consisted of nine self-help modules. The modules were based on the book *Unlearn your Pain* (36), which presents a self-help version of EAET, but a new series of modules was written in Swedish for internet delivery. These modules were reviewed by the founders of EAET (ML and HS) and judged as consistent with EAET principles and techniques. As shown in Table 1, the modules covered three main components: (1) psychoeducation and increased awareness of pain, the body-mind connection, and pain neuroscience; (2) defense recognition and anxiety regulation; and (3) emotional exposure. In Modules 1 and 2, patients were educated about how the brain generates the perception of pain and other somatic symptoms by integrating peripheral nociception with cognitions and emotions, and that the brain's neural pathways are sensitized by emotions and stressful life events. Patients were encouraged to look for possible connections between stressful life events and somatic symptoms by mapping out the relationships between the onset of progress of somatic symptoms and the events of their lives. In Module 3, the concept of defenses was introduced, especially the importance of identifying the defense of turning anger inwards/self-criticism. Patients also were taught how to identify their defense and develop a self-soothing capacity with self-compassion meditation exercises. Modules 4–8 comprised the main component of the treatment—emotional exposure. In Modules 4–6, patients were encouraged to do expressive writings exercises, with a specified protocol following psychodynamic principles in which patients first processed anger, guilt about anger, and then sadness and love. In Modules 7 and 8, the emotional exposure was continued in real relationships where the participants learned to be more emotionally expressive and assertive. This was accomplished by, for example, encouraging patients to write unsent letters where all feelings were expressed and then later using the content of the letter to seek out important relationships and express the important parts in real relationships. Finally, in Module 9, patients reviewed the changes they made, focused on insights they learned, and planned for their futures.

**Table 1:** Structure and content of internet-based emotional awareness and expression therapy.

**EAET**	**Main theme**	**Main treatment component**	**Main homework**
Module 1–2	Psychoeducation on the body-mind connection	Psychoeducation, insight and awareness	Look for a possible connection between somatic symptoms and stressful life-events
Module 3	Turning anger inwards/self-critical thoughts	Defense recognition and anxiety regulation	Identify defense and develop a self-soothing capacity. Learn to do self-compassion meditation exercises
Module 4–6	Expressive writing as emotional processing	Emotional exposure	Do expressive writing exercises and process anger, guilt about anger, sadness and love
Module 7–8	Learn to be emotionally expressive and assertive	Emotional exposure	Express feelings in important relationships and balance assertion with intimacy
Module 9	Summary and lessons learned	Insight and awareness	Summarize insights and plan for the future

In addition to the self-help modules, patients were given therapist support and guidance. All contacts between patients and therapists were written text messages in a secure internet environment. Specifically, the therapists checked in with patients once per week and gave written feedback on homework assignments. In their feedback, therapists used not only supportive interventions but also encouraged patients to deepen their emotional exposure and help them better regulate their anxiety. Therapists accomplished these goals in several ways. If patients expressed only anger and not sadness, for example, therapists encouraged patients to write about the avoided feeling. They encouraged anxiety regulation by having patients name their emotions or made summaries of links between feelings, bodily symptoms/anxiety, and defenses. Less often, therapists used traditional psychodynamic interpretations of unconscious processes. Despite using the internet, therapists tried to build a therapeutic alliance by giving personalized answers to any messages from the patients within 24 h. It is known that a strong therapist-client alliance can be established in internet-based treatments (37), even in short-term therapy such as this one (38). There were seven therapists in the study, six of whom were psychology students in training to become licensed psychologists, and the other (first author) had worked as a clinical psychologist for over 12 years. All therapists were given one training session by RJ on how to be an internet therapist, and two training sessions by HS on how to conduct EAET specifically.

### Primary Outcome Measure

Given that EAET targets somatic symptoms, the Patient Health Questionnaire-15 [PHQ-15; (39)] was used as the primary outcome measure. The PHQ-15 has been found to be a moderately reliable questionnaire for the detection of somatoform disorders in primary care (40) and in the general population (41). It consists of 15 somatic symptoms which patients' rate “not bothered at all” (0), “bothered a little” (1), or “bothered a lot” (2). Total scores range from 0 to 30, and scores of 5, 10, and 15 represent cut-offs for mild, moderate, and severe levels of somatic symptoms. Fair to good psychometric properties have been demonstrated, with good internal consistency Cronbach's α = 0.80 (39). The PHQ-15 has been validated in the Swedish population, showing similar psychometric properties (33). In this sample, internal consistency at baseline was acceptable, with α = 0.60. For this measure, we also calculated the minimally clinically important difference (MCID) as a reduction in the PHQ-15 score of at least 2.3 points (42).

### Secondary Outcome Measures

The Patient Health Questionnaire-9 [PHQ-9; (39)] assessed depressive symptom severity. This self-report measure consists of nine items rated 0–3, and total scores range from 0 to 27. The PHQ-9 has good psychometric properties, including an internal consistency in the range of Cronbach's α = 0.86 – 0.89 (39). In this sample, internal consistency at baseline was good: α = 0.76.

The Generalized Anxiety Disorder-7 scale [GAD-7; (43)] assessed anxiety symptom severity. This self-rated measure consists of seven items rated 0–3, and total scores range from 0 to 21. Internal consistency is excellent (Cronbach's α = 0.92) (39). In this sample, internal consistency at baseline was good, with α = 0.85.

The Post-Traumatic Stress Disorder Checklist version 5 [PCL-5; (44)] is a 20-item self-report measure of DSM-5 PTSD symptoms. Items are rated from 0 to 4, and total scores range from 0 to 80. The PCL-5 is psychometrically sound, with strong internal consistency (Cronbach's α = 94) (44). In this sample, internal consistency at baseline was excellent, with α = 0.92.

The Sheehan Disability Scale [SDS; (45)] assessed functional impairment in three domains: work/school, social and family life. Each domain is assessed by one item, rated from 0 to 10; total scores range from 0 to 30, and higher scores indicate more functional impairment. The SDS has good internal consistency (α = 0.77) (46). In this sample, internal consistency at baseline was good, with α = 0.74.

### Feasibility Measures

The 32-item Negative Effect Questionnaire [NEQ; (47)] assessed negative effects following treatment. The scale assesses five domains: dependency (e.g., “I think I developed a dependency on my therapist”), symptoms (e.g., “I experienced more unpleasant feelings”), hopelessness (e.g., “I started thinking that the issue I was seeking help for could not be made any better”), failure/stigma (e.g., “I experienced lower self-esteem), and quality (e.g., “I felt that the quality of the treatment was poor”). Patients report whether specific items occurred during treatment, and if so, how negative the effect was (rated from 0 to 4), and whether the effect was attributed to “the treatment I received” or “other circumstances.” Psychometric properties have been found to be strong for the NEQ (Cronbach's α = 0.95). The NEQ was administered at the end of treatment.

The Credibility Expectancy Questionnaire [CEQ; (48)] consists of three credibility and three expectancy items regarding the treatment a patient is participating in. Total scores range from 0 (not at all credible/no expectancy for improvement) to 100 (very credible/large expectancy for improvement). The CEQ has good psychometric properties with a standardized alpha of *r* = 0.85 (for both scales) (48). Patients completed the CEQ at the beginning of week 3/module 3, after they had learned about the treatment content and rational.

In addition to questionnaires, patient had the opportunity to describe what had been most helpful and most challenging aspects of the treatment.

### Feasibility Criteria

Several rather rigorous feasibility criteria were set for this study, including criteria for treatment adherence and credibility, attrition, adverse events, and satisfaction with treatment. Following previous research, adherence was deemed sufficient if the proportions of completed modules in the treatment where > 70% (49). Moreover, we aimed for a high level of treatment credibility (>70% of patients) and satisfaction with treatment (>80% of patients would recommend this treatment to others); these indices were calculated by using the credibility subscale in CEQ (where the question of treatment satisfaction is derived). Given that ~34% of people usually drop-out of internet-delivered treatment (50), an attrition rate lower than 35% was deemed acceptable. In prior studies of psychiatric populations, negative effects on the individual items of NEQ have been reported by 5.5–65.2% of patients (51) rendering it difficult to set a specific criterion; nevertheless, we proposed that negative effects reported in each of the five NEQ domains by fewer than 10% of participants was acceptable.

### Data Analyses

Dependent samples *t*-tests were performed to assess the statistical significance of changes from pre-treatment to post-treatment and pretreatment to follow-up. Effect sizes and 95% confidence intervals of changes between assessment points were calculated as within-group effect size Cohen's *d*, accounting for the correlation between measurement points (52). Effect sizes were categorized according to Cohen's proposal, where small, medium, and large effect sizes are *d* ≥ 0.20, 0.50, and 0.80, respectively (53). Differences between treatment completers and non-completers were assessed using independent samples *t*-tests. All calculations were conducted using Jamovi (54).

To determine whether the feasibility criterion of adherence was met (i.e., at least 70% completed treatment modules), the total number of modules completed for all patients was divided by the total number of modules available. To calculate treatment credibility, the total score of all patients' ratings was divided by the maximum score, multiplied by 100. To calculate negative effects from NEQ, the percentage of all items in each domain were summed and then divided by number of items in that domain.

## Results

### Recruitment and Participant Characteristics

**Figure 1:**
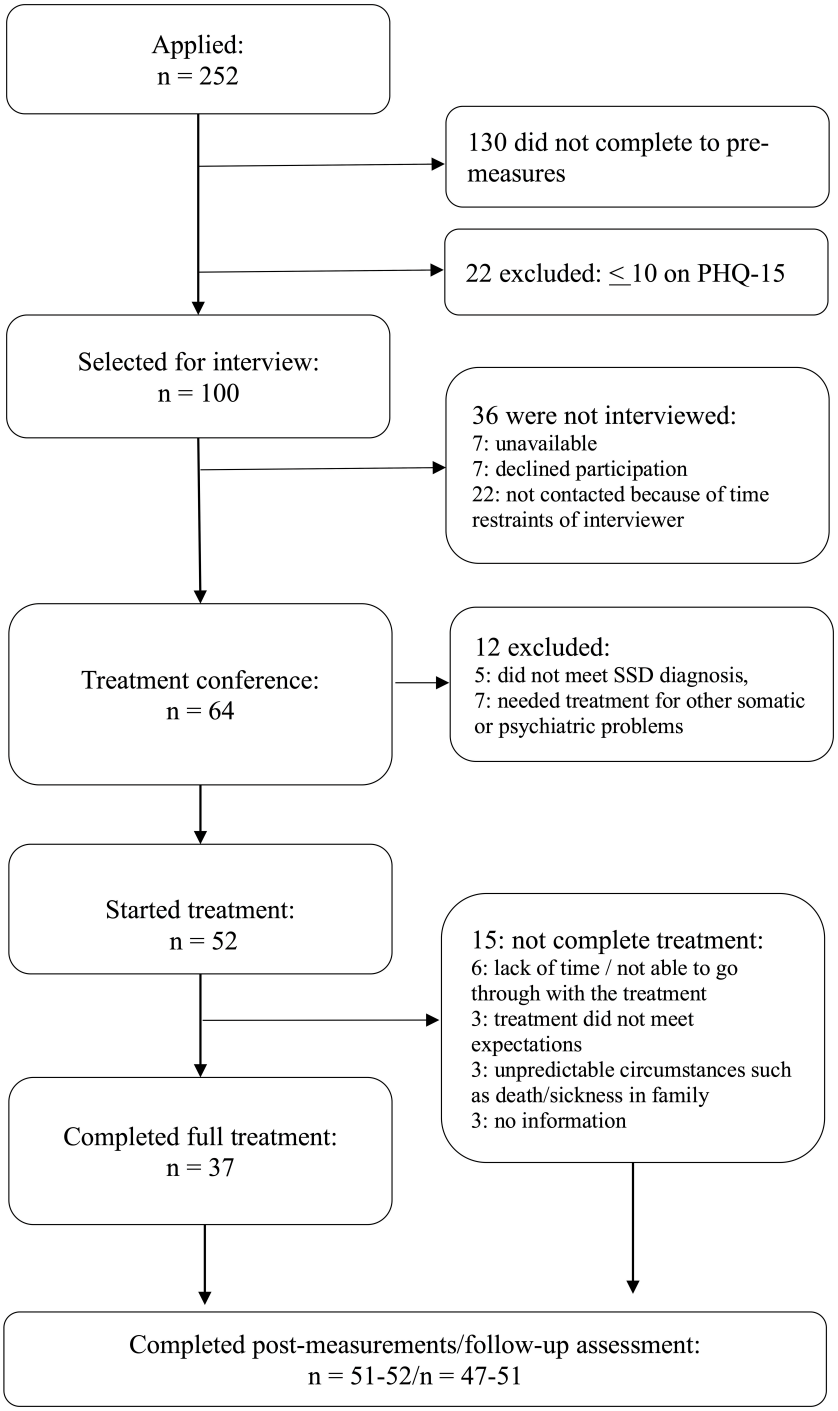
Study flowchart.

The recruitment and trial flow are shown in Figure 1. A total of 252 people initially expressed interest in participating, 122 completed the initial screening, 64 had a telephone interview, and 52 were included in the study.

Of these 52 patients, 37 (71%) completed all nine treatment modules, whereas the other 15 patients completed zero (*n* = 1), one (*n* = 1), two (*n* = 1), three (*n* = 5), six (*n* = 3), or seven (*n* = 4) modules. Figure 1 notes several reasons for ending therapy prematurely; however, the 15 treatment non-completers did not differ from the 37 treatment completers on any pre-treatment measure (all *t*'s < 1.382, all *p*'s > 0.173).

Study attrition was low. At post-treatment, all 52 patients (i.e., including those who did not complete all modules) completed the primary outcome (PHQ-15), and the secondary outcomes were completed by 51 (GAD-7, SDS, PCL-5) or 52 (PHQ-9) patients. At 4-month follow-up, all patients except one completed the PHQ-15, and secondary measures were completed by 47 (SDS, PCL-5) or 49 (PHQ-9, GAD-7) patients. Given the low attrition, analyses were conducted on available data, with no replacement of missing values.

As shown in Table 2, the 52 patients were almost exclusively women (96.2%), with a mean age of 49.6 years. Most participants were married or co-habiting (61.5%) and had less than college or university education (55.8%). Nearly a third were on sick leave (30.8%), and two-thirds (*n* = 35) had ongoing pharmacological treatment, most of whom (*n* = 27; 77%) were taking medications for anxiety, depression or sleep disturbances, pain (*n* = 20, 57%), or other medical conditions (*n* = 13, 28.6%).

**Table 2:** Demographic description of the participants.

**Gender: *N* (%)**.	**Female: 50 (96.2%)**
Age: Mean (*SD*); min/max.	49.6 (11.9); 28 – 72
Marital status: Married or co- habiting.	32 (61.5%)
Educational level: College or university:	23 (44.2%)
Sick leave: 100%	8 (15.4%)
50%	7 (13.5%)
25%	1 (1.9%)
NO	36 (69.2%)
Prior psychological treatment: YES	45 (86.5%)
Ongoing psychological treatment: YES	10 (19.2%)
Ongoing pharmacological treatment: YES	35 (67.3%)
**Comorbidity**	
Depression	26 (50%)
Panic disorder	10 (19.2%)
Agoraphobia	6 (11.5%)
PTSD	5 (9.6%)
Social anxiety disorder	4 (7.7%)
Generalized anxiety disorder	2 (3.8)
Illness anxiety disorder	1 (1.9%)
Obsessive compulsion disorder	1 (1.9%)
ADHD	1 (1.9%)
No (psychiatric comorbidity)	8 (15.4%)
Yes	44 (84.6%)
One or two (psychiatric diagnosis)	34 (65.4%)
Three or more	10 (19.2%)

The majority of participants had been in previous psychological treatment (86.5%), and a minority had ongoing supportive psychological treatment (19.2%). There was substantial psychiatric comorbidity; over 80% of the sample had an additional psychiatric diagnosis: 50% had comorbid depression, 20% had panic disorder, and 20% had three to six other diagnoses. All patients reported pain as either a major or minor part of their condition (Table 3). The most common somatic condition reported by patients was fibromyalgia (42.3% of patients), and more than half of the sample reported at least two somatic conditions.

**Table 3:** Self-reported somatic problems of patients.

**Self-reported diagnosis**	**Number with reported somatic problem (% of patients reporting the problem)**
Fibromyalgia	22 (42.3%)
IBS	13 (25%)
Chronic pain (e.g., chronic back pain, dyspepsia, vulvodynia, etc.)	13 (25%)
Exhaustion disorder (ICD code 438.A)	12 (23%)
Migraine	8 (15%)
“Other” (reporting symptoms such as pain, fatigue, etc.)	8 (15%)
Ehlers-Danlos Syndrome (with judged sensitization)	5 (9.6%)
Tinnitus	4 (7.7%)
Chronic Fatigue Syndrome	4 (7.7%)
Whiplash	2 (3.8%)
**Number of above diagnoses: 1**	24 (46.2%)
2	18 (34.6%)
3	5 (9.6%)
4	5 (9.6%)

### Feasibility

Tables 4, 5 summarize the feasibility criteria used in the study and whether the pilot study met these criteria. As can be seen in Table 5, all criteria were met, except for negative effects. Treatment attrition rates were lower (29%) than the pre-specified 35%. Adherence was high given that on average, patients completed 85% of modules, whereas the criterion was set at 70%. Criteria for credibility (>70%) and satisfaction (>80%) were met. Regarding negative effects, in four of the five domains, acceptable negative effects were reported; no more than around 10% of participants reported dependency, hopelessness, stigma/failure, or lack of quality with treatment. However, symptom increase potentially related to the treatment was more frequent, reported in average by 29%. Moreover, during treatment, one critical event occurred: a patient developed suicidal ideation. This patient discontinued treatment and was monitored and found to be stable at follow up.

**Table 4:** Feasibility outcomes.

	**Definition**	**%**
Attrition	<35% attrition	37 of 52 followed-through all modules, (29% attrition)
Negative effects	<10% report any negative effect in all domains in NEQ	Met for four out of five domains (see Table 5)
Adherence	>70% completed modules	397/468 = 85% completed modules
Credibility	>70% find treatment credible	7.2/9 = 80%
Satisfaction	>80% would recommend treatment	7.29/9 = 81%

**Table 5:** NEQ domains (total % of reported negative effect).

Symptom increase (e.g., “unpleasant memories resurfaced”)	28.6%
Lack of quality of treatment (e.g., “I felt that the quality of the treatment was poor”	7.84%
Increased hopelessness (e.g., “I started thinking that the issue I was seeking help for could not be made any better”)	10.8%
Dependency on treatment (e.g., “I think I developed a dependency on my therapist”	10.8%
Experiencing failure or stigma (e.g., “I experienced lower self-esteem”)	3.9%

When describing their experiences of specific treatment components, many patients reported that psychoeducation, work on defenses and anxiety regulation (i.e., targeting self-criticism with self-compassion), and writing about stressful life events were especially helpful. The treatment was deemed challenging and time-consuming, and confronting pain and painful emotions in interpersonal relationships was difficult.

### Treatment Effects

As shown in Table 6, a large within-group reduction for the primary outcome of somatic symptoms (PHQ-15) was found *(d* = 1.13; 95% CI: 0.84 – 1.47) at the end of treatment. Almost one-quarter of the sample (23.1% or 12/52) achieved a 50% or larger reduction in somatic symptoms from pre-treatment. The large within–group reduction in somatic symptoms was fully maintained (even slightly increased) at 4-month follow-up *(d* = 1.19; 95% CI: 0.88 – 1.56), when 26.9% (14/52) of the participants had a 50% or larger symptom reduction on the PHQ-15. Based on another metric, the majority of patients–37 of 52 (71.2%) at post-treatment and 36 of 52 (69.2%) at follow-up reached a MCID in reduction in PHQ-15 scores.

**Table 6:** Means, SDs, effect sizes (Cohen's d), and MCID for outcomes across assessment times.

**Measure**	**Pre-treatment mean (*SD*)**	**Post-treatment mean (*SD*)**	**4-month Follow-up mean (*SD*)**	**Pre – post effect Cohen's *d* (95% CI)**	**Pre – follow-up effect Cohen's *d* (95% CI)**
PHQ-15 (0-30)	16.0 (3.70)	11.0 (4.95)	11.0 (4.48)	1.13 [0.84; 1.47]	1.19 [0.88; 1.56]
PHQ-9 (0-27)	13.1 (4.49)	10.4 (6.33)	9.18 (5.54)	0.50 [0.21; 0.81]	0.79 [0.48; 1.12]
GAD-7 (0-21)	9.31 (4.97)	6.84 (5.27)	6.94 (5.71)	0.44 [0.16; 0.75]	0.46 [0.19; 0.75]
PCL-5 (0-80)	54.2 (17.4)	47.7 (17.6)	42.2 (18.5)	0.33 [0.01; 0.66]	0.66 [0.33; 1.01]
SDS (0-30)	21.6 (5.96)	19.0 (8.60)	15.2 (10.2)	0.38 [0.10; 0.67]	0.80 [0.47; 1.12]
MCID 2.3->		37/52 (70.2%)	36/52 (69.2%)		

Analyses indicated small to moderate magnitude reductions in secondary outcomes at post-treatment, including anxiety (GAD-7; *d* = 0.44), depression (PHQ-9; *d* = 0.50), and trauma-related symptoms (PCL-5; *d* = 0.33). At 4-month follow-up, these effects were either maintained (GAD-7; *d* = 0.46) or increased further (PHQ-9; *d* = 0.79; PCL-5; *d* = 0.66). The treatment also significantly increased patients' ability to take part in social and family life (SDS) at post-treatment (*d* = 0.38) which increased substantially at follow-up (*d* = 0.80).

## Discussion

This pilot study is the first to examine the feasibility and effectiveness of an internet-administered EAET for adult patients with SSD and centralized somatic symptoms (SSD-CS). A large within-group reduction in somatic symptoms was observed at post-treatment, and this effect was fully maintained at 4-month follow-up. The majority (71.2%) of the patients achieved a minimally clinical important reduction in somatic symptoms—and over one-quarter (26.9%) achieved at least 50% reduction—at follow-up. These results further strengthen the evidence base for EAET and suggest that the treatment can be effectively delivered in a guided, internet-based self-help format. Moreover, according to rather rigorous feasibility criteria, the internet-delivered treatment was deemed to be feasible, as treatment completion, credibility, and satisfaction were well within acceptable levels.

The uncontrolled design of this study precludes making certain conclusions about the effects of this intervention. However, the substantial reduction in somatic symptoms appears to be larger than what has been reported in a meta-analysis of internet-administered, primarily CBT interventions for samples similar to ours. Vugts et al. (19) reported only a small reduction in somatic symptoms at follow-up (*d* = −0.18) for such interventions when compared to passive controls. In addition, in a meta-analysis of CBT for fibromyalgia (which was the most prevalent condition in our sample), Bernardy et al. (22) found that only 13.3% of patients received a 50% symptom reduction at the end of treatment, whereas it was observed for 23.1% of the patients in this study and slightly more at follow-up. Moreover, the results of this study seem comparable to results obtained in face-to-face trials of EAET (17, 18, 29). For example, the Yarns et al. (18) study of group-based EAET for chronic musculoskeletal pain found a within-group reduction for pain severity of *d* = 0.76 at post treatment, whereas the current study found a somewhat larger reduction in somatic symptoms (*d* = 1.13). We realize that such cross-study comparisons are limited, of course, and trials comparing I-EAET to I-CBT and to face-to-face EAET are needed to adequately test the superiority or non-inferiority of I-EAET.

In addition to reductions in somatic symptoms, we observed small to medium magnitude reductions in anxiety, depression, and trauma symptoms, which were maintained or increased at 4-month follow-up. In addition, patients' daily behavioral functioning was improved, especially at 4-month follow-up, where a large effect was obtained. The large improvements in somatic vs. emotional/psychiatric symptoms are consistent with previous studies of EAET, which often show greater reductions in somatic than in psychiatric symptoms. Why this occurs remains unclear, but it has been proposed that because EAET activates memories of stressful life events and avoided emotions, EAET may make patients more aware of their emotional distress and more likely to report it rather than somatic symptoms such as pain (23). Emotional symptoms may take longer to remit, and the current study offered some support for this, in that depression and trauma symptoms continued to decrease over the 4-month follow-up period. Yet the reduction in psychiatric symptoms, particularly anxiety and trauma symptoms, did not reach the level of reduction in somatic symptoms. Further research on change processes within EAET treatment may further elucidate this issue.

Based on rather rigorous criteria, the I-EAET treatment was deemed to be feasible with respect to treatment completion as well as treatment credibility and satisfaction. For internet-delivered treatments, drop-out/non-completion is rather common, with estimates as high as 34.2%, and higher than in face-to-face studies (50). The non-completion rate found in this study, which was based on the very conservative requirement that all modules needed to be completed, was lower than this criterion and, therefore, interpreted to be acceptable for an internet-delivered treatment. Even though patients with persistent physical symptoms do not drop out of treatment at a higher rate than patient with most other psychiatric disorders (55), depression is known to increase dropout (55, 56). Given that half the sample in this study had a comorbid depressive diagnosis, which is consistent with the results of Alda et al. (57), the acceptability of the low drop-out rate in this study is further underscored. Previous research has set a 70% completion rate of modules to be an acceptable level of adherence (49), and adherence in this study was well above that number. Moreover, the treatment was judged both highly credible and acceptable, further strengthening the feasibility of this study.

The question of whether a treatment has negative effects is important, and a fear of negative effects of psychological treatment for persistent physical symptoms have been voiced (58). We found that few patients reported dependency, hopelessness, stigma/failure, or lack of quality with treatment. A meaningful minority, however, reported an increase in symptoms during treatment, including unpleasant memories resurfacing during treatment. Experiencing such memories, however, may not be a negative effect of I-EAET, given that this treatment specifically targets accessing and processing avoided emotions related to stressful life events and conflicts. Thus, this finding may reflect successful treatment engagement. Patients expressed almost exclusively positive interest in the psychoeducation of the body-mind connection (module 1–2 of the treatment), and most patients followed through with daily writing exercises and meditations on how to be less self-critical and be more able to identify and express complex feelings of anger, guilt, sadness, and love (modules 3–6). Many patients, however, struggled with targeting and confronting triggers of emotional or pain avoidance (modules 7–8); generally, however, emotional processing of painful memories was reported by patients to be challenging yet helpful.

There are various limitations to this study. Notably, the lack of a randomized control group limits concluding that it was the treatment, *per se*, that was responsible for the observed benefits; passage of time, history or maturation, and repeated testing could have contributed to the effects. However, SSD is a chronic and relatively unchanging condition, so findings that 70% achieved a MCID in somatic symptom reduction, and over 25% achieved at least 50% symptom reduction suggest a true treatment effect. Regardless, a controlled trial is needed to determine the effects of I-EAET specifically, and whether this approach might be superior to other internet-delivered treatments. Of course, the sample was relatively small, and a larger sample is indicated. Another limitation is that all outcomes were exclusively self-report, which has known limitations, and the use of experimental measures such as quantitative sensory testing, behavioral indicators such as physical functioning and down-time, and health care costs would be important to validate the self-reports and evaluate the breadth of this treatment's effects. The primary outcome measure, PHQ-15, had only acceptable internal consistency, which might be due to chance or reflect the heterogeneity of the sample in this study. To be able to capture true effects of the treatment, it might be necessary to use more specific questionnaires of, for example, pain and fatigue rather than a broad instrument such as PHQ-15. Although the diagnosis of SSD was used, we did not specifically assess all of the B criteria for SSD as outcome measures; we note, however, that we did include measures of dysfunctional emotions—depression and anxiety—as well as behavior—disability, in our assessment battery.Generalizability of study findings might be limited in that the sample was almost exclusively female, experienced with psychological therapy, relatively educated, employed, and self-selected into the study based on their interest in investigating possible emotional factors contributing to somatic symptoms. How this treatment fares among patients in routine care, who are more disabled or less educated, or who are skeptical of the relevance of psychological factors remains to be tested. Moreover, although SSD was diagnosed, the sample was restricted to patients with moderate physical symptoms (>10 point in PHQ-15) and with centralized physical symptoms. The results of the study, therefore, cannot be generalized to the broader population of SSD patients.

The model underlying EAET proposes that better treatment outcomes for persistent physical symptoms may require addressing the consequences of trauma and increasing emotional awareness and emotional processing of trauma and conflict. In contrast, most CBT protocols for persistent physical symptoms do not focus on these precipitating and perpetuating factors, but instead change unhelpful cognitions (e.g., by reappraisal), down-regulate arousal (e.g., by relaxation training), and increase daily functioning (e.g., by activity pacing) (15, 16, 59). It is noteworthy that some recent CBT models have increased the focus on emotional regulation. For example, Boersma et al. (60) found that exposure-based CBT with emotion-regulation skills training had a better effect on depression and pain interference than traditional I-CBT. Kleinstäuber et al. (61) found that CBT enriched with emotional regulation training was more beneficial than traditional CBT for patients with a co-morbid mental disorder. Although several studies of CBT for patients with IBS have not found similar moderation (62, 63), it is possible that the relatively elevated rates of comorbid mental disorders in our sample contributed to the success of I-EAET. Together with recent successful studies of CBT based on exposure to both external and internal stimuli (64), the field is increasingly recognizing the importance of exposure to bodily sensations and the accompanying emotions, which are facets of both EAET and more newly developed CBT protocols. More generally, such findings strengthen the conclusion that emotional factors should be directly addressed in treatment of persistent physical symptoms.

In conclusion, this preliminary study supports both the feasibility and efficacy of I-EAET in an adult population of patients with SSD and centralized somatic symptoms with high psychiatric comorbidity. Although uncontrolled, this study suggests that EAET can substantially reduce somatic symptoms, presumably by addressing avoided emotional experiences and engaging in emotional processing. Over one-quarter of the patients in this study had a substantial and durable reduction in somatic symptoms. Nevertheless, controlled trials comparing I-EAET to a basic control condition and eventually to a bona fide alternative intervention, such as I-CBT, are needed to demonstrate the specific efficacy of this intervention and whether it is superior to other approaches. Although greater emotional expression is linked to better psychotherapy outcomes (65), research also is needed to test hypothesized mechanisms by examining the content of patients' engagement. Future research should also focus on identifying those patients who benefit the most from I-EAET and ways to reach a broader range of patients.

## Data Availability Statement

The datasets presented in this article are not readily available because participants did not consent to this. Therefore, the dataset is available on reasonable requests as deemed by the principal investigator of the study. Requests to access the datasets should be directed to the Principal Investigator. Requests to access the datasets should be directed to Robert Johansson, robert.johansson@psychology.su.se.

## Ethics Statement

The studies involving human participants were reviewed and approved by Swedish Ethical Review Authority (Dnr 2019-03317). The patients/participants provided their written informed consent to participate in this study.

## Author Contributions

DM and RJ designed the study, with ML in an advisory role and conducted the statistical analysis. DM, R-MW, and JE were three of the therapists in the study. RJ and HS supervised the therapists. DM wrote the first draft of the manuscript. ML contributed to interpretation of data analysis and revising the manuscript. All authors contributed to revising the manuscript and accepting its final version.

## Conflict of Interest

HS is the owner of Mind Body Publishing, a company that sells books written by HS for patients dealing with mind body symptoms and for professionals who treat such patients. BL is shareholder of DahliaQomit AB, a company specializing in online psychiatric symptom assessment, and Hedman-Lagerlöf och Ljótsson psykologi AB, that licenses a treatment manual for irritable bowel syndrome on a commercial basis. The remaining authors declare that the research was conducted in the absence of any commercial or financial relationships that could be construed as a potential conflict of interest.
